# Nrf2 activation mediates tumor-specific hepatic stellate cells-induced DIgR2 expression in dendritic cells

**DOI:** 10.18632/aging.102554

**Published:** 2019-12-13

**Authors:** Yun-Hong Xia, Zhen Lu, Shou-Min Wang, Li-Xia Hu

**Affiliations:** 1 Department of Oncology, The Fourth Affiliated Hospital, Anhui Medical University, Hefei, China; 2 Department of General Surgery, The Fourth Affiliated Hospital, Anhui Medical University, Hefei, China; 3 Department of Oncology, Hefei Hospital, Anhui Medical University, Hefei, China

**Keywords:** hepatic stellate cells (tHSCs), hepatocellular carcinoma (HCC), DIgR2, dendritic cells, Nrf2 signaling

## Abstract

Our previous studies discovered that tumor-specific hepatic stellate cells (tHSCs) induced dendritic cell-derived immunoglobulin receptor 2 (DIgR2) expression in bone marrow-derived dendritic cells (mDCs), inhibiting splenic T cell activation. The current study aims to explore the underlying mechanism of DIgR2 expression by focusing on Nrf2 (nuclear-factor-E2-related factor 2) signaling. We show that tHSCs co-culture induced significant Nrf2 signaling activation in mDCs. The latter was evidenced by Nrf2-Keap1 disassociation, Nrf2 protein stabilization, accumulation and nuclear translocation. Expression of Nrf2-dependent genes, including *heme oxygenase-1 (HO-1)* and *NAD(P)H:quinone oxidoreductase 1 (NQO1)*, were detected in tHSCs-co-cultured mDCs. Importantly tHSCs-induced DIgR2 expression was blocked by Nrf2 shRNA or knockout (KO, by CRISPR/Cas9 method). Conversely, forced activation of Nrf2, by Keap1 shRNA or the Nrf2 activators (3H-1,2-dithiole-3-thione and MIND4-17), induced significant DIgR2 expression. tHSCs stimulation induced reactive oxygen species (ROS) production in mDCs. Conversely, ROS scavengers inhibited tHSCs-induced ROS production, Nrf2 activation and DIgR2 expression in mDCs. Significantly, tHSCs inhibited production of multiple cytokines (CD80, CD86 and IL-12) in mDCs, reversed by Nrf2 depletion. Moreover, Nrf2 shRNA or KO attenuated splenic T cell inhibition by tHSCs-stimulated mDCs. Together, we conclude that Nrf2 activation mediates tHSCs-induced DIgR2 expression in mDCs.

## INTRODUCTION

Hepatocellular carcinoma (HCC) is a major health threat and a primary cause of cancer-associated human mortalities [1–3]. HCC’s incidence and the mortality rate are rising [4–6]. Tumor immunity has become one research focus for HCC [7–9]. Dendritic cells (DCs) are antigen-presenting cells (APC) in tumor immunity, initiate key immune responses [10, 11]. The proper activation of DCs is important for efficient anti-tumor immunity [10, 11]. Conversely, depletion or inhibition of DCs could lead to pro-cancer tumor environments [10, 11]. DIgR2, or dendritic cell-derived immunoglobulin receptor 2, is a member of IgSF inhibitory receptor, inhibiting DC-induced antigen-specific T-cell responses [12]. DCs-derived DIgR2 directly binds to T cells, suppressing T-cell-mediated tumor immunity [12].

Hepatic stellate cells (HSC) are the primary source of extracellular matrix in the fibrogenesis process [13–15]. Recent literatures have implied that tumor-specific hepatic stellate cells (tHSCs) promote HCC tumorigenesis and progression [13–15]. tHSCs, characterized by α-smooth muscle actin expression, can differentiate into myofibroblasts [13–15]. The tumor-promoting tHSCs express intercellular adhesion molecule 1 (ICAM-1) and vascular cell adhesion molecule 1 (VCAM-1), among others, to facilitate migration, invasion and proliferation of tumor cells [13–15]. Our recent studies have discovered that tHSCs induces DIgR2 expression in bone marrow-derived dendritic cells (mDCs) to inhibit T cell functions [16, 17]. The underlying signaling mechanisms are not fully understood.

Under basal conditions, nuclear-factor-E2-related factor 2 (Nrf2) binds to its suppressor protein, Kelch-like ECH-associated protein 1 (Keap1) [18–21]. The latter is a Cullin 3 (Cul3) E3 ubiquitin ligase [18–21], dictating Nrf2 ubiquitination and proteasomal degradation [18–21]. Agents or stimuli modifying cysteine residues of Keap1 will lead to Keap1-Nrf2 disassociation, Nrf2 protein accumulation and nuclear translocation. It will eventually lead to transcription and expression of ARE (anti-oxidant response element)-dependent antioxidant genes [18–21]. The results of this study will show that Nrf2 activation mediates tHSCs-induced DIgR2 expression in mDCs.

## RESULTS

### tHSCs co-culture induces Nrf2 signaling activation in mDCs

**Figure 1:**
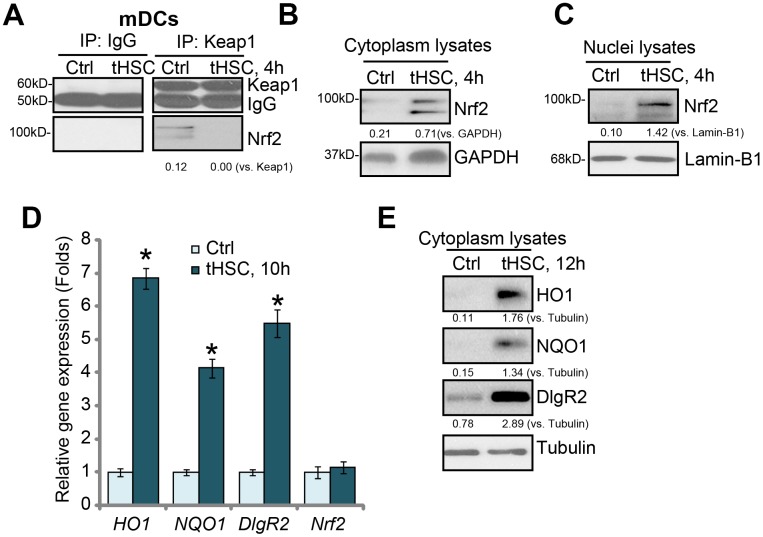
**tHSCs co-culture induces Nrf2 signaling activation in mDCs.** The bone marrow-derived dendritic cells (mDCs) were co-cultured with/without tumor HSCs (tHSCs; mDCs to tHSCs ratio, 20: 1) for applied time, Nrf2-Keap1 association was tested by the co-immunoprecipitation (“Co-IP”) (**A**); Expression of listed proteins in cytoplasm lysates (**B** and **E**) and nuclei lysates (**C**) were tested by Western blotting; Expression of listed mRNAs was tested by qPCR (**D**). Listed proteins were quantified and normalized to the loading control (**A**–**C**, **E**). Lamin-B1 is a nuclear marker protein (**C**). “Tubulin” stands for the loading control β-Tubulin (Same for all Figures). Data are presented as the mean ± standard deviation (n=5). “Ctrl” stands for mDCs only (no tHSCs stimulation). * P < 0.05 vs. “Ctrl” group. The experiments in this figure were repeated four times, and similar results were obtained.

Our previous studies have shown that tHSCs co-culture induces DIgR2 expression in bone marrow-derived DCs (mDCs) [16, 17]. The current study aims to test the potential involvement of Nrf2 signaling in the process. First, we tested whether tHSCs co-culture activated Nrf2 signaling activation in mDCs. As described [16, 17], mDCs were co-cultured with primary tHSCs (mDCs to tHSCs ratio, 20: 1). A co-immunoprecipitation (“Co-IP”) assay was performed to test the association between Nrf2 and its suppressor protein Keap1 [22, 23]. As shown, Nrf2 immunoprecipitated with Keap1 in the untreated control mDCs (Figure 1A). Following co-culture with tHSCs, Nrf2 was disassociated with Keap1 (Figure 1A). Furthermore, Nrf2 protein was accumulated in the cytoplasm of mDCs (Figure 1B). Additionally, analyzing nuclear lysate proteins in mDCs confirmed that stabilized Nrf2 translocated to cell nuclei following tHSCs stimulation (Figure 1C). These results show that tHSCs co-culture induced Nrf2-Keap1 disassociation, Nrf2 protein accumulation and nuclear translocation in mDCs (Figure 1A–1C).

Following nuclear translocation, Nrf2 will bind to ARE to promote transcription and expression of multiple anti-oxidant and detoxifying enzymes, including *heme oxygenase-1 (HO-1)* and *NAD(P)H:quinone oxidoreductase 1 (NQO1)* [22, 23]. We show that *HO1* and *NQO1* mRNA (Figure 1D) and protein (Figure 1E) levels were significantly increased in tHSCs-stimulated mDCs. In line with our previous studies [16, 17], *DlgR2 mRNA* and protein levels were increased as well in mDCs with tHSCs stimulation (Figure 1D and 1E). The *Nrf2* mRNA levels were however unchanged (Figure 1D), indicating that Nrf2 protein upregulation was due to post-transcriptional regulation (Nrf2-Keap1 disassociation). These results suggest that tHSCs co-culture induced Nrf2 signaling activation in mDCs.

### Nrf2 activation is required for tHSCs-induced DIgR2 expression in mDCs

**Figure 2:**
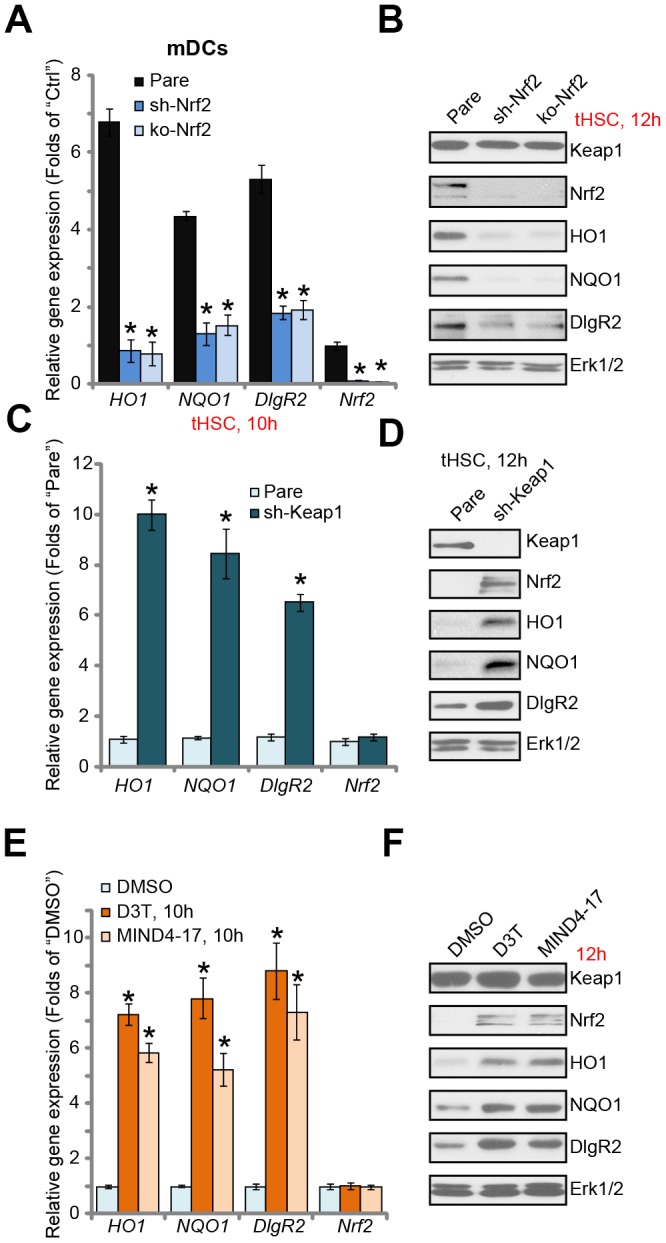
**Nrf2 activation is required for tHSCs-induced DIgR2 expression in mDCs.** The stable bone marrow-derived dendritic cells (mDCs), with Nrf2 shRNA (“sh-Nrf2”) or Nrf2 KO construct (“ko-Nrf2”), as well as the parental control mDCs (“Pare”), were co-cultured with tumor HSCs (tHSCs; mDCs to tHSCs ratio, 20: 1) for applied time, expression of listed genes was shown (**A** and **B**). Expression of listed genes in stable mDCs with Keap1 shRNA (“sh-Keap1”) or the parental control mDCs (“Pare”) was shown (**C** and **D**). Control mDCs were treated with 3H-1,2-dithiole-3-thione (D3T, 25 μM), MIND4-17 (10 μM) or vehicle control (0.25% DMSO) for applied time, listed genes were shown (**E** and **F**). Data are presented as the mean ± standard deviation (n=5). “Ctrl” stands for mDCs only. * P < 0.05 vs. “Pare” cells (A and **C**). * P < 0.05 vs. “DMSO”-treated cells (**E**). The experiments in this figure were repeated three times, and similar results were obtained.

Next, we tested the functional activity of Nrf2 activation in tHSCs-induced DIgR2 expression in mDCs. To block Nrf2 activation, genetic strategies [24, 25] were utilized. First, the Nrf2 shRNA lentiviral particles were added to mDCs, with puromycin selection the stable cells were established. Additionally, the CRISPR/Cas9 gene editing method was employed to complete knockout (KO) Nrf2 in mDCs (see Methods). Testing *Nrf2* mRNA and protein expression confirmed that Nrf2 was depleted in stable mDCs with Nrf2 shRNA or Nrf2 KO construct, even after tHSCs stimulation (Figure 2A and 2B). tHSCs-induced HO1 and NQO1 expression, the Nrf2 target genes [26, 27], was blocked by Nrf2 shRNA or KO (Figure 2A and 2B). Importantly, Nrf2 shRNA or KO reversed tHSCs-induced *DIgR2* mRNA and protein expression in mDCs (Figure 2A and 2B). These results indicate that Nrf2 activation is required for tHSCs-induced DIgR2 expression in mDCs.

We further hypothesized that forced activation of Nrf2 will induce DIgR2 expression in mDCs. Therefore, the Keap1 shRNA lentiviral particles were added to mDCs. Following puromycin selection, the stable cells were established. In Keap1 shRNA-expressing mDCs, expression of HO1, NQO1, and more importantly DIgR2, was significantly increased (Figure 2C and 2D). Accumulation of Nrf2 protein, but not *Nrf2* mRNA, was detected (Figure 2C and 2D). Thus Keap1 silencing induced Nrf2 activation and DIgR2 expression in mDCs. To further support our results, we show that two established Nrf2 activators, 3H-1, 2-dithiole-3-thione (D3T) [28, 29] and MIND4-17 [30, 31], induced Nrf2 protein stabilization as well as HO1, NQO1 and DIgR2 expression in mDCs (Figure 2E and 2F). Collectively, these results show that Nrf2 activation is required for tHSCs-induced DIgR2 expression in mDCs.

### tHSCs-induced Nrf2 activation and DIgR2 expression in mDCs is associated with reactive oxygen species (ROS) production

**Figure 3:**
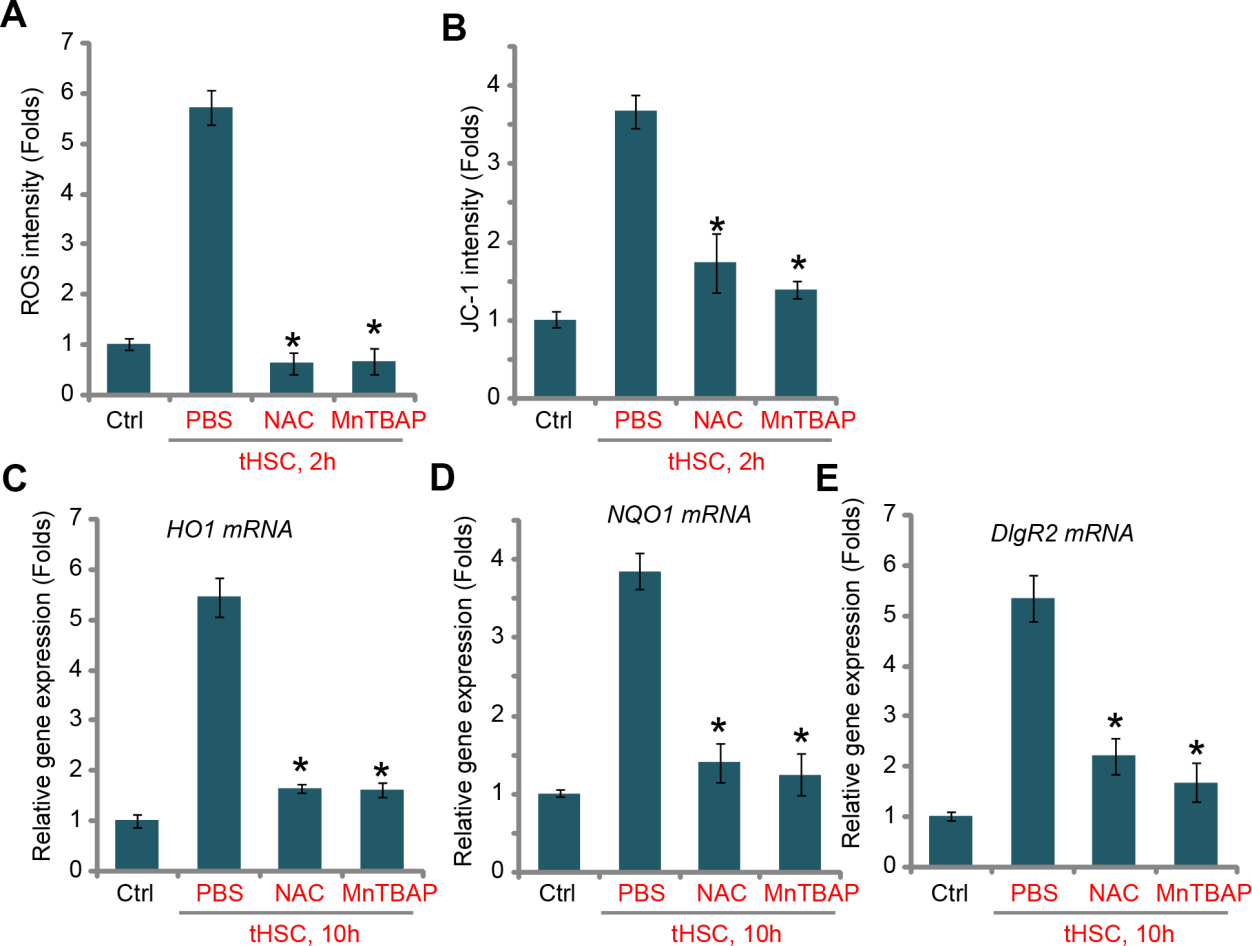
**tHSCs-induced Nrf2 activation and DIgR2 expression in mDCs is associated with reactive oxygen species (ROS) production.** The bone marrow-derived dendritic cells (mDCs) were pretreated for 1h with N-acetylcysteine (NAC, 500 μM) or MnTBAP (40 μM), followed by co-culture with tumor HSCs (tHSCs; mDCs to tHSCs ratio, 20: 1) for applied time, the ROS contents and mitochondrial depolarization were tested by H2DCFDA dye (**A**) and JC-1 dye (**B**) assays, respectively; Relative expression of listed genes were tested by qPCR assay (**C**–**E**). “Ctrl” stands for mDCs only. “PBS” stands for vehicle control PBS. Data are presented as the mean ± standard deviation (n=5). * P < 0.05 vs. “PBS” group. The experiments in this figure were repeated four times, and similar results were obtained.

ROS production and oxidative stress will induce Keap1 acetylation and Keap1-Nrf2 disassociation, causing Nrf2 stabilization and activation [22, 32]. Thus, we tested whether tHSCs co-culture could induce ROS production in mDCs. Using the H2DCFDA fluorescent dye assay, our results found that ROS levels were significantly increased in tHSCs-stimulated mDCs (Figure 3A), where the H2DCFDA fluorescent intensity increased over five folds of control level (Figure 3A). Moreover, mitochondrial depolarization, evidenced by JC-1 green fluorescent intensity increase, was detected in mDCs with tHSCs co-culture (Figure 3B). Two well-known ROS scavengers, N-acetylcysteine (NAC) and Mn (III) tetrakis (4-benzoic acid) porphyrin (MnTBAP) [33, 34], blocked tHSCs-induced ROS production and mitochondrial depolarization (Figure 3A and 3B). Significantly, tHSCs-induced expression of *HO1* (Figure 3C), *NQO1* (Figure 3D), and *DIgR2* (Figure 3E), was largely inhibited by NAC and MnTBAP in mDCs. These results indicate that ROS production is essential for tHSCs-induced Nrf2 activation and DIgR2 expression in mDCs.

### tHSCs-induced Nrf2 activation in mDCs contributes to inhibition on cytokines

**Figure 4:**
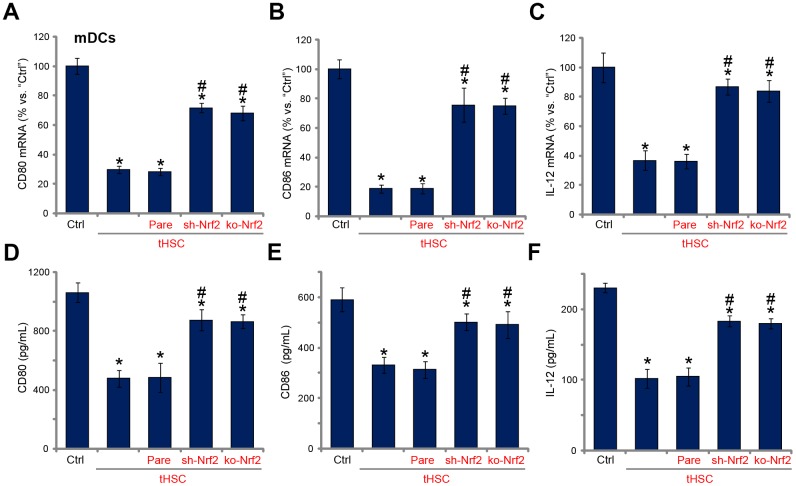
**tHSCs-induced Nrf2 activation in mDCs contributes to inhibition on cytokines.** The stable bone marrow-derived dendritic cells (mDCs), with Nrf2 shRNA (“sh-Nrf2”) or Nrf2 KO construct (“ko-Nrf2”), as well as the parental control mDCs (“Pare”), were co-cultured with tumor HSCs (tHSCs) for 72h, *mRNA expression* (qPCR assay, **A**–**C**) and protein contents (in conditional medium, ELISA, **D**–**F**) of listed cytokines were tested. Data are presented as the mean ± standard deviation (n=5). “Ctrl” stands for mDCs only. * P < 0.05 vs. “Ctrl”. ^#^ P < 0.05 vs. “Pare” group. The experiments in this figure were repeated four times, and similar results were obtained.

Our previous studies have shown that tHSCs co-culture significantly inhibited production of multiple cytokines in mDCs. Such actions were abolished with DIgR2 depletion. Since Nrf2 activation is required for DIgR2 expression, we proposed that Nrf2 depletion should abolish tHSCs-induced inhibition on cytokines in mDCs. In line with our previous findings, in mDCs expression of CD80 (one key surface co-stimulatory molecule, Figure 4A), CD86 (another surface co-stimulatory molecule, Figure 4B) and IL-12 (DC activation marker cytokine, Figure 4C) was decreased following tHSCs co-culture. Significantly, Nrf2 shRNA or KO alleviated tHSCs-mediated inhibition of the cytokines in mDCs (Figure 4A–4C). ELISA results further show that productions of CD80 (Figure 4D), CD86 (Figure 4E) and IL-12 (Figure 4F) were reduced in tHSCs-stimulated mDCs. Such actions were inhibited with Nrf2 silencing or KO (Figure 4D–4F). These results imply that tHSCs-induced Nrf2 activation in mDCs contributes to inhibition on cytokines.

### Nrf2 activation mediates splenic T cell inhibition by tHSCs-stimulated mDCs

**Figure 5:**
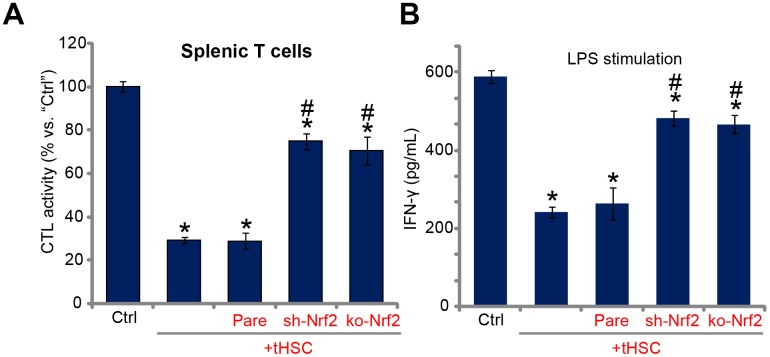
**Nrf2 activation mediates splenic T cell inhibition by tHSCs-stimulated mDCs.** The stable bone marrow-derived dendritic cells (mDCs), with Nrf2 shRNA (“sh-Nrf2”) or Nrf2 KO construct (“ko-Nrf2”) as well as the parental control mDCs (“Pare”) were co-cultured with tumor HSCs (tHSCs) for 24h; Stimulated mDCs were then co-cultured with splenic T cells; OVA-II peptide CTL assay activity (after 24h, **A**) and LPS (100 ng/mL)-induced IFN-γ production (after 24h, **B**) were tested. Data are presented as the mean ± standard deviation (n=5). “Ctrl” stands for mDCs only. * P < 0.05 vs. “Ctrl”. ^#^ P < 0.05 vs. “Pare” group. The experiments in this figure were repeated three times, and similar results were obtained.

Studies from our group [16, 17] and others have demonstrated that DC-derived DIgR2 binds to its receptor in T cells, suppressing T cell functions. Using the described protocol [16, 17], the splenic T cells were co-cultured with mDCs (T cells to mDCs ratio, 20:1). Ovalbumin II (OVA-II) peptide cytotoxic T lymphocyte (CTL) assay results demonstrated that tHSCs-stimulated mDCs can significantly inhibit the CTL activity of splenic T cells. Furthermore, lipopolysaccharides (LPS) -induced interferon-γ (IFN-γ) production in splenic T cells was largely inhibited after co-culture of tHSCs-stimulated mDCs (Figure 5B). Importantly, Nrf2 shRNA or KO in mDCs (see Figure 2) almost reversed splenic T cell inhibitions by tHSCs-stimulated mDCs (Figure 5A and 5B). These results suggest that Nrf2 activation is important for splenic T cell inhibitions by tHSCs-stimulated mDCs.

## DISCUSSION

Immune evasion of HCC cells [7–9] is vital for cancer progression [9, 35]. tHSCs could be an important cause of T-cell hypo-responsiveness in HCC. Studies have shown that B7-H1 (a key member of the B7 family of co-stimulatory molecules) upregulation in tHSCs can lead to increased ligation of PD-1 receptor on T-cells. This will lead to T cell apoptosis and suppression of T-cell-mediated tumor cell apoptosis [36–38]. Our previous study has shown that tHSCs significantly induced DIgR2 expression in mDCs. The latter is a key negative regulator of DC-initiated T-cell response [12].

The present study shows that following tHSCs stimulationNrf2 signaling was activated in mDCs. Nrf2 cascade activation was evidenced by Nrf2-Keap1 disassociation, Nrf2 protein stabilization and accumulation, as well as Nrf2 nuclear translation and transcription of ARE-dependent genes (*HO1* and *NQO1*). Importantly, Nrf2 activation is required for tHSCs-induced DIgR2 expression in mDCs. Nrf2 shRNA or CRISPR/Cas9-mediated Nrf2 KO blocked DIgR2 expression in tHSCs-treated mDCs. Conversely, forced activation of Nrf2, through Keap1 shRNA or Nrf2 activators (D3T and MIND4-17), induced *DIgR2* expression in mDCs. Therefore, DIgR2 could be a novel Nrf2-dependent gene in mDCs.

ROS will induce Keap1 acetylation, causing Nrf2-Keap1 disassociation and Nrf2 signaling activation [22, 39]. One novel finding of the current study is that tHSCs co-culture induced significant ROS production and oxidative stress in mDCs. In tHSCs-treated mDCs ROS production and mitochondrial depolarization were detected. Importantly, ROS production should be the direct cause of Nrf2 activation and DIgR2 expression in mDCs. Two ROS scavengers, NAC and MnTBAP, not only blocked tHSCs-induced ROS production, but also inhibited Nrf2 activation and DIgR2 expression in mDCs. The underlying mechanisms of ROS-mediated Nrf2 activation in tHSCs-treated mDCs need further characterizations.

DIgR2 is an important member of IgSF inhibitory receptor [40–42]. DigR2 inhibits DC-initiated antigen-specific T-cell responses [12, 40–42]. It has two immuno-receptor tyrosine-based inhibitory motifs in the cytoplasmic region [12]. DigR2 associates with tyrosine phosphatase protein SHP1. DC-derived DIgR2 binds directly to T cells, causing T-cell inhibition and hypo-responses [12]. DIgR2 inhibition, contrarily, can boost DC-provoked T-cell responses [12]. We have previously shown that tHSCs-stimulated mDCs induced T-cell hypo-responsiveness, causing inhibited CTL activity and reduced IFN-γ production by LPS [16, 17]. In the present study, we show that Nrf2 shRNA or KO also reversed splenic T cell inhibition by tHSCs-stimulated mDCs. Therefore, Nrf2 activation is important for splenic T cell inhibition by tHSCs-stimulated mDCs, although the signaling mechanism may warrant further characterizations. In summary, our results show that Nrf2 activation mediates tHSCs-induced DIgR2 expression in mDCs.

## MATERIALS AND METHODS

### Chemicals, reagents and antibodies

Antibodies were obtained from Abcam (Cambridge, MA). From Invitrogen-Life Technologies (Grand Island, NY) the cell culture reagents were obtained. Puromycin, lipopolysaccharide (LPS), 3H-1,2-dithiole-3-thione (D3T), N-acetylcysteine (NAC) and MnTBAP [Mn (III) tetrakis (4-benzoic acid) porphyrin Chloride] were purchased from Sigma-Aldrich (St. Louis, MO). MIND4-17 was provided by Dr. Wang [31]. All the verified primers for quantitative real-time PCR (qPCR) assay were purchased from OriGene Technologies (Rockville, MD).

### Rat HCC tumor model

As previously described [43, 44], MRH HCC cells [43, 44] were initially injected to the flanks of the buffalo rats. Within four weeks the xenograft tumors were established. HCC tumors were cut into small pieces (2×1×1 mm^3^), transplanted to rat livers [43]. Using a ultrasound method in situ tumor growth was confirmed. The animal protocols were approved by Anhui Medical University’s IACUC and Ethics Board.

### Primary culture of tHSCs

As previously described [16, 17, 43, 44], tHSCs were derived from the in situ HCC tissues from the buffalo rats. HCC tissues were first subjected to perfusion [43, 44]. Afterwards, truncated HCC tissues were further digested [43, 44]. Thereafter, single cell suspensions were established, subjecting to purification by centrifugation through a 8% Nycodenz (Axis-Shield PoC) gradient. The acquired tHSCs were cultured in FBS containing DMEM with necessary supplements [43, 44]. Via a trypan blue exclusion assay cell viability >95% was verified. A desmin immuno-staining assay was performed to verify the purity of tHSCs (>95%) [45].

### Primary culture of mDCs and tHSCs-mDCs co-culture

As previously described [43, 46], the bone marrow was isolated. The red blood cells were abolished, and bone marrow cells were cultured in the described medium [43, 44] plus GM-CSF (Sigma) and IL-4 [16, 17]. The non-adherent cells were acquired from the proliferating cell clusters, harvested, washed, resuspended and cultured in medium described [46]. For the co-culture experiments, in each well 2.5×10^5^ mDCs were incubated with 1.25×10^4^ tHSCs (20: 1, mDCs to HSCs).

### Primary culture of spleen T cells

As described previously [43, 44], the minced rat spleens were filtered. Splenocytes were isolated from erythrocytes via centrifugation of the cell suspension on a Ficoll gradient (Histopaque 1083, Sigma) [47]. The cells were layered onto the top of the gradient in a 10-ml Falcon tube, followed by centrifugation at 800×g for 20 min at room temperature. Lymphocytes (mainly T-cells) were collected, washed and cultured as described [43, 44].

### Co-culture of spleen T cells with mDCs and *in vitro* T cell function detection

As reported [16], spleen T cells were co-cultured with mDCs (T cells/mDCs ratio = 20:1). An OVA (323-339) peptide assay kit was utilized to test cytotoxic T lymphocyte (CTL) activity [12]. T cells were also treated with LPS (100 ng/mL, Sigma), supernatants were collected after 24h for enzyme-linked immunosorbent assay (ELISA) detection of interferon-γ (IFN-γ).

### RNA extraction and qPCR

Trizol reagents (Promega, Madison, WI) were utilized for RNA extraction. A SYBR Green PCR kit (Applied Biosystems, Foster City, CA) was utilized for reverse transcription. qPCR assays were performed using the previously described protocol [16, 17]. The product melting temperature was calculated via using a melt curve analysis. We utilized the 2^−ΔΔ*C*t^ method to quantify targeted gene expression, using *glyceraldehyde-3-phosphatedehydrogenase* (*GAPDH)* as the reference gene and internal control.

### Western blotting

Lysates (30 μg proteins of each treatment in each lane) were separated by a 10% SDS-PAGE gel, transferred to a polyvinylidene difluoride (PVDF) blot (Millipore, Temecula, CA) [48]. After incubation in PBST with 10% non-fat milk, the blot was incubated with applied primary and secondary antibodies. To visualize the targeted band, we utilized an enhanced chemiluminescence (ECL, Roche, Shanghai, China) detection kit. An ImageJ software was applied to quantify the band intensity (total gray). The nuclear lysates were isolated by using a previously described protocol [49].

### Co-immunoprecipitation (Co-IP) assay

As described [24], following co-culture with tHSC, 500 μg of total cell lysates of mDCs were first pre-cleared by using A/G Sepharose (“Beads”, Sigma-Aldrich). Thereafter, an anti-Keap1 antibody (Abcam) was included in the pre-cleared lysates overnight, followed by adding back the protein A/G Sepharose for 2h. Keap1-immunoprecipitated Nrf2 was then tested via Western blotting.

### Reactive oxygen species (ROS) detection

H2DCFDA (dichlorofluorescin diacetate), a fluorogenic dye, measures hydroxyl, peroxyl and other ROS activity. In brief, following co-culture with tHSCs, mDCs were incubated with H2DCFDA (5 μM, Abcam) dye for 45 min under dark. H2DCFDA absorbance was detected by a fluorescence spectroscopy with excitation and emission at 495 nm and 529 nm, respectively.

### Mitochondrial depolarization assay

Tetraethylbenzimidazolylcarbocyanine iodide (JC-1) is a cationic dye that accumulates in energized mitochondria. It forms green monomers after mitochondrial depolarization. In brief, following co-culture with tHSC, mDCs were incubated with JC-1 dye (10 μM, Abcam) dye for 30 min under dark at the room temperature. At the test wavelength of 530 nm JC-1 green fluorescence absorbance was tested.

### Short hairpin RNA (shRNA)-mediated stable knockdown of Nrf2 or Keap1

The Nrf2 lentiviral particles or the Keap1 shRNA lentiviral particles were purchased from Shanghai Genechem (Shanghai, China), added to mDCs for 12h. Cells were then cultured in fresh complete medium (with 10% FBS), following by selection with puromycin (2.0 μg/mL) for another 48h. In the stable cells over 95% of knockdown of target protein (Nrf2 or Keap1) was detected.

### Nrf2 knockout

The single-guide RNA (sgRNA) targeting Nrf2 was sub-cloned into a lenti-CRISPR-GFP-puro construct. The Nrf2 knockout (KO) construct was transfected to mDCs by Lipofectamine 2000. mDCs were further subjected to selection with puromycin (2.0 μg/mL) for 48h. In the stable cells Nrf2 KO was verified by Western blotting and qPCR.

### Statistical analysis

All statistical analyses were performed by using SPSS 18.0 software. Values are expressed as the mean ± standard deviation (SD). P < 0.05 was considered to indicate a statistically significant result.
